# Wrist-Based Photoplethysmography Assessment of Heart Rate and Heart Rate Variability: Validation of WHOOP

**DOI:** 10.3390/s21103571

**Published:** 2021-05-20

**Authors:** Clint R. Bellenger, Dean J. Miller, Shona L. Halson, Gregory D. Roach, Charli Sargent

**Affiliations:** 1 Alliance for Research in Exercise, Nutrition and Activity (ARENA), Allied Health and Human Performance, University of South Australia, Adelaide 5000, Australia; 2 South Australian Sports Institute, Adelaide 5000, Australia; 3 The Appleton Institute for Behavioural Science, Central Queensland University, Adelaide 5043, Australia; d.j.miller@cqu.edu.au (D.J.M.); greg.roach@cqu.edu.au (G.D.R.); charli.sargent@cqu.edu.au (C.S.); 4 School of Behavioural and Health Sciences, Australian Catholic University, Brisbane 4014, Australia; Shona.Halson@acu.edu.au

**Keywords:** autonomic nervous system, agreement, electrocardiogram

## Abstract

Heart rate (HR) and HR variability (HRV) infer readiness to perform exercise in athletic populations. Technological advancements have facilitated HR and HRV quantification via photoplethysmography (PPG). This study evaluated the validity of WHOOP’s PPG-derived HR and HRV against electrocardiogram-derived (ECG) measures. HR and HRV were assessed via HR and HRV were assessed via WHOOP 2.0 and ECG over 15 opportunities during October–December 2018. WHOOP-derived pulse-to-pulse (PP) intervals were edited with WHOOP’s proprietary filter, in addition to various filter strengths via Kubios HRV software. HR and HRV (Ln RMSSD) were quantified for each filter strength. Agreement was assessed via bias and limits of agreement (LOA), and contextualised using smallest worthwhile change (SWC) and coefficient of variation (CV). Regardless of filter strength, bias (≤0.39 ± 0.38%) and LOA (≤1.56%) in HR were lower than the CV (10–11%) and SWC (5–5.5%) for this parameter. For Ln RMSSD, bias (1.66 ± 1.80%) and LOA (±5.93%) were lowest for a 200 ms filter and WHOOP’s proprietary filter, which approached or exceeded the CV (3–13%) and SWC (1.5–6.5%) for this parameter. Acceptable agreement was found between WHOOP- and ECG-derived HR. Bias and LOA in Ln RMSSD approached or exceeded the SWC/CV for this variable and should be interpreted against its own level of bias precision.

## 1. Introduction

Resting heart rate (HR) quantification and monitoring have been common in exercise physiology research and practice for centuries [1]. Additionally, HR variability (HRV), a sophisticated derivative of HR, has been quantified to provide insight into cardiac modulation by the parasympathetic and sympathetic divisions of the autonomic nervous system (ANS) [2]. Given the integral role of the ANS to all physiological function, including those related to exercise and training [3], the body’s ability to tolerate or adapt to an exercise stimulus may be inferred by examining ANS responsiveness [3]. Consequently, HRV has been used to infer training tolerance or readiness to perform exercise in athletes [4,5].

Advancements in HR monitor technology, namely the first wireless HR monitor [6], have facilitated frequent and accurate HR quantification. However, this technology is reliant on the wireless communication of the heart’s electrical activity from an elastic electrode chest strap to relevant receivers, and such reliance on chest straps can be inconvenient and problematic. Regarding HR and HRV assessment for the day-to-day monitoring of readiness to perform specifically, compliance is challenged by wearing a chest strap during daily recordings [7]. Consequently, HR and HRV assessments that occur without a chest strap, such as photoplethysmography (PPG), are advantageous.

PPG detects changes in pulsatile blood flow between the heart’s systole and diastole via LED-emitted light at the wrist, fingertip or earlobe [8]. The LED light illuminates the skin, while a photodetector quantifies the intensity of the light reflected back from the skin [7]. Since blood volume is acutely increased following cardiac systole (which obstructs the LED light and reduces the intensity of the reflected light) and decreased during cardiac diastole (increasing the intensity of reflected light), the heart’s rhythm can be detected via PPG [7].

Calculation of HR and HRV from pulse-to-pulse (PP) intervals quantified via PPG is not a novel concept, having been utilised in research as historically as 1938 [9]. However, its contemporary application has been facilitated by technological advancements in commercially available HR monitors. PPG validation studies demonstrate acceptable agreement in HR quantification at rest [8,10] and during sleep [11]. Similarly, PPG-derived resting HRV (and specifically the root mean square of successive beat to beat (BB) interval differences; RMSSD) demonstrates acceptable agreement assessed at the earlobe and fingertip at rest [7,12,13,14,15,16,17] and during sleep [18].

WHOOP 2.0 is a wearable biosensor that quantifies HR and HRV (in the form of RMSSD) via wrist-based PPG. Uniquely, however, WHOOP quantify these measures during slow-wave sleep (SWS) [19], which it is able to determine with moderate accuracy [20]. Given that SWS is thought to be important for physiological recovery from exercise [21,22,23], and HRV is considered a marker of physiological recovery [4,5], HRV assessment during SWS may quantify the degree of physiological recovery facilitated by this sleep stage, and thus may be used in the day-to-day monitoring of training status by practitioners. Additionally, WHOOP subsequently utilise HR and HRV measures (along with sleep duration) in an algorithm to predict a “Recovery Score” out of 100% [19]. This Recovery Score may be used to individually guide training prescription as a measure of readiness to perform. Given the novelty of WHOOP for assessing wrist-based PPG-derived HR and HRV, in addition to its unique dependence on measuring SWS in its quantification of HR and HRV, this study aimed to evaluate the agreement between WHOOP-derived HR and HRV and gold-standard assessment via ECG during SWS episodes.

## 2. Materials and Methods

### 2.1. Participants

Six healthy, young adults (male: *n* = 3; female: *n* = 3; age: 22.9 ± 3.4 years) participated in this study. Participants were excluded if they reported any existing medical conditions or sleep disorders, or had a recent history of shift work and/or transmeridian travel. This study was approved by the Central Queensland University Human Research Ethics Committee.

### 2.2. Experimental Overview

Data collection occurred in October–December 2018, concurrently with a larger pre-existing sleep study which has not yet been published. Data were collected over three consecutive sleep opportunities at the Appleton Institute of Behavioural Science, Central Queensland University, which contains two co-located, purpose-built accommodation suites that are sound attenuated, free from external environmental cues and can simultaneously house a total of six participants with private bedrooms and bathrooms. Participants wore a WHOOP 2.0 unit (CB Rank, Greater Boston, New England) on their non-dominant wrist during sleep opportunities at the end of day 1 (2300–0800), end of day 2 (0300–1200) and during day 4 (1430–2130). Agreement between WHOOP and ECG-derived HR and HRV was evaluated through four time- and SWS stage-matched analyses (Table 1). Firstly, agreement between time-matched WHOOP- and ECG-derived HR and HRV was assessed during the final WHOOP-derived SWS episode in line with the technology’s ecological use, and across a range of data editing filters to determine the extent to which WHOOP-derived PP intervals need to be edited for erroneous PP intervals, including WHOOP’s proprietary filter. Secondly, to determine whether the accurate identification of the final SWS episode impacts upon the agreement between WHOOP- and ECG-derived HR and HRV (given that WHOOP has only moderate sensitivity to accurately identifying sleep stage [20]), WHOOP- and ECG-derived HR and HRV were also quantified during the final polysomnography (PSG)-derived SWS episode for comparison. Additionally, to provide insight into the impact of misrepresentation of true SWS periods by WHOOP on HR and HRV, two SWS stage-matched analyses were conducted. Consequently, the third analysis assessed agreement between ECG-derived HR/HRV (i.e., true HR/HRV) during PSG-derived SWS episodes (i.e., true SWS) and WHOOP-derived HR/HRV during WHOOP-derived SWS episodes. The fourth analysis assessed the agreement between WHOOP-derived HR/HRV during PSG-derived SWS episodes and WHOOP-derived HR/HRV during WHOOP-derived SWS episodes as a means of determining the impact of SWS misrepresentation on WHOOP-derived measures.

### 2.3. Sleep Stage Identification

To acquire WHOOP strap sleep data, researchers manually entered the start and end times of each sleep opportunity into the WHOOP smart phone application. The manufacturer then provided data in 30 s epochs for wake, light sleep, SWS and rapid eye movement (REM) sleep. PSG data were recorded directly to data acquisition, storage and analysis systems (Grael, Compumedics; Victoria, Australia). Brain, eye and muscle activity were quantified from electrodes attached to the face and scalp of participants, including three electroencephalography electrodes (i.e., C4-M1, F4-M1, O2-M1), two electro-oculograms (i.e., left/right outer canthus) and a submental electromyogram. PSG records were manually scored (in 30 s epochs) by a registered and experienced polysomnographic technician in compliance with standard criteria [24]. Time in bed during each sleep opportunity was arranged into wake, non-rapid eye movement sleep (non-REM; stage 1 [S1], stage 2 [S2] and SWS) and rapid eye movement (REM) sleep. Cardiac activity was assessed via two ECG electrodes (left-positive and right-negative) recorded using the aforementioned Grael PSG system. The negative electrode was placed three centimetres below the right clavicle, positioned on the torso parallel to the right leg. The positive electrode was positioned on the left side of the torso parallel to the left hip and leg, between either the fifth, sixth, or seventh intercostal spaces on the lower left side of the rib cage.

### 2.4. Heart Rate and Heart Rate Variability Calculation

Using both the WHOOP- and PSG-derived sleep staging data for each sleep opportunity, the final five minutes of the final SWS episodes were identified. If the final sleep stage was less than five minutes in duration, the preceding stages were identified until a five-minute stage was found. Subsequently, time-matched PP and RR intervals were extracted from WHOOP- and ECG-derived files, respectively, for each SWS episode and analysed using WHOOP’s proprietary filter and HRV analysis software (Kubios HRV Analysis, version 2.0 beta 1, Biomedical Signals Analysis Group, University of Kuopio, Finland). To determine the degree of PP interval editing required to facilitate the best agreement between WHOOP- and ECG-derived measures, Kubios’s default filters (i.e., “Low”, “Medium”, “Strong” and “Very Strong”—equivalent to 400, 300, 200 and 100 ms editing thresholds, respectively), in addition to no filter (i.e., “None”) and WHOOP’s proprietary filter, were separately applied to WHOOP-derived PP intervals and recorded for analysis. For HRV analysis, RMSSD and its natural logarithm transformation (i.e., Ln RMSSD) were recorded for analysis.

### 2.5. Statistical Analysis

Data were analysed using SPSS (IBM Corp. IBM SPSS Statistics for Windows, Version 25.0. Armonk, NY, USA) and presented as the mean ± 95% confidence intervals. Agreement between WHOOP- and ECG-derived measures of HR and HRV was determined through absolute and percentage mean bias (WHOOP minus ECG), absolute and percentage limits of agreement (LOA) and intra-class correlation (ICC). ICCs were evaluated as: 0.0–0.1, trivial; 0.1–0.3, small; 0.3–0.5, moderate; 0.5–0.7, large; 0.7–0.9, very large; 0.9–1.0, nearly perfect [25].

For the parameters of HR, RMSSD and Ln RMSSD, separate two-way (filter strength × SWS quantification method [i.e., WHOOP vs. PSG]) repeated measures ANOVAs determined statistically significant differences in ECG and WHOOP-derived values, and in bias between filter strengths for each SWS quantification method. As it was not possible to statistically compare LOA between filters and SWS quantification method, mean residuals were calculated as a measure of variability about the bias and compared via separate two-way (filter strength × SWS quantification method) repeated measures ANOVAs for HR, RMSSD and Ln RMSSD. Individual residuals were calculated as the square root of the squared difference between the individual value and the mean value for both absolute and percent bias. Statistical significance was set at *p* < 0.05.

For values of HRV, the filter strength resulting in the smallest bias and smallest LOA, in addition to WHOOP’s proprietary filter, was subsequently used to compare differences in percent bias and percent residuals between analytical method (i.e., RMSSD vs. Ln RMSSD) via two-way ANOVA.

Effect sizes ([ES] with 95% confidence intervals) between variables of interest were calculated using pooled standard deviation. Threshold values for ES were ≤0.2 (trivial), >0.2 (small), >0.6 (moderate), >1.2 (large), >2.0 (very large), and >4.0 (extremely large) [25].

## 3. Results

Of the 18 opportunities for data collection, HR and HRV data from three sleep opportunities were lost due to equipment malfunction and/or experimenter error. Thus, data from 15 sleep opportunities were available for comparison.

### 3.1. Filter Analysis

**Figure 1:**
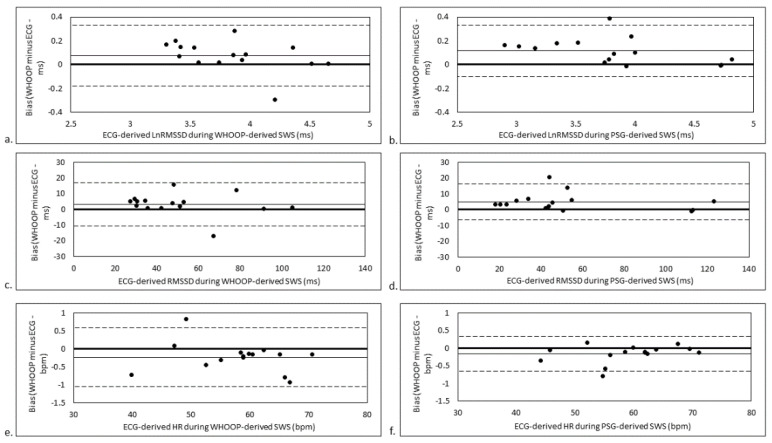
Agreement between (**a**) ECG-derived Ln RMSSD and WHOOP-derived Ln RMSSD during WHOOP-derived SWS, (**b**) ECG-derived Ln RMSSD and WHOOP-derived Ln RMSSD during PSG-derived SWS, (**c**) ECG-derived RMSSD and WHOOP-derived RMSSD during WHOOP-derived SWS, (**d**) ECG-derived RMSSD and WHOOP-derived RMSSD during PSG-derived SWS, (**e**) ECG-derived HR and WHOOP-derived HR during WHOOP-derived SWS and (**f**) ECG-derived HR and WHOOP-derived HR during PSG-derived SWS. Thin continuous line represents mean bias. Dashed lines represent mean bias ± limits of agreement. bpm, beats per minute; ECG, electrocardiogram; HR, heart rate; Ln RMSSD, natural logarithm of the root mean square of successive BB interval differences; ms, milliseconds; PSG, polysomnography; RMSSD, root mean square of successive BB interval differences; SWS, slow-wave sleep.

With regard to WHOOP’s proprietary filter, trivial but statistically significant biases were found between WHOOP- and ECG-derived Ln RMSSD across both SWS quantification methods (percent bias ≤3.25 ± 1.53%; bias as ES ≥0.18 ± 0.16; *p* ≤ 0.04; LOA ≤6.59%; Figure 1a,b), and RMSSD during PSG-derived SWS (percent bias = 12.74 ± 6.53%; bias as ES = 0.14 ± 0.09; *p* = 0.005; LOA = 25.31%; Figure 1d), but not RMSSD during WHOOP-derived SWS (percent bias = 8.54 ± 6.65%; bias as ES = 0.13 ± 0.15; *p* = 0.10; LOA = 25.75%; Figure 1c).

Regarding the BB editing filters applied in Kubios, a Strong filter (equivalent to a 200 ms editing threshold) applied to WHOOP-derived PP intervals resulted in the smallest percent bias and smallest percent LOA across both SWS quantification methods for RMSSD (percent bias ≤8.39 ± 6.70%; bias as ES ≤0.08 ± 0.15; LOA ≤27.26%) and Ln RMSSD (percent bias ≤2.30 ± 1.69%; bias as ES ≤0.14 ± 0.17; LOA ≤6.98%) (Supplementary Tables S1 and S2).

In comparison to Kubios’s Strong filter, the bias between WHOOP and ECG values was greater when edited using WHOOP’s proprietary filter during PSG-derived SWS across both RMSSD (ES = 0.51 ± 0.24; *p* = 0.04) and Ln RMSSD values (ES = 0.34 ± 0.14; *p* = 0.03). Percent LOAs were similar when WHOOP-derived PP intervals were edited using Kubios’s Strong filter compared to WHOOP’s proprietary filter across both SWS quantification methods for RMSSD and Ln RMSSD, and analysis of residuals indicated no statistical differences (ES ≤0.50 ± 0.59; *p* ≥ 0.12).

With regard to HR, there was a trivial but statistically significant bias between WHOOP-derived HR edited by WHOOP’s proprietary filter and ECG-derived HR during both WHOOP- and PSG-derived SWS (ES ≥−0.03 ± 0.02; percent bias ≥−0.39 ± 0.38%; *p* ≤ 0.04; Figure 1e,f). For all other filters, there were trivial and non-statistically significant biases between WHOOP- and ECG-derived HR (ES ≤0.006 ± 0.023; *p* ≥ 0.59). LOA for all filters across both SWS quantification methods were ≤1.56% and ICCs were almost perfect (r = 1.00 [95% confidence interval range 0.99 to 1.00]; Supplementary Table S3).

### 3.2. Analytical Method Analysis

For both Kubios’s Strong filter and WHOOP’s proprietary filter, percent bias for measures of Ln RMSSD were smaller than measures of RMSSD (ES ≤−0.73 ± 0.61; *p* ≤ 0.03) with the exception of Kubios’s Strong filter during WHOOP-derived SWS which trended toward statistical significance (ES = −0.56 ± 0.60; *p* = 0.09). Similarly, percent residuals for measures of Ln RMSSD were smaller than measures of RMSSD (ES ≤−1.23 ± 0.60; *p* ≤ 0.001).

### 3.3. SWS Stage-Matched Analyses

**Figure 2:**
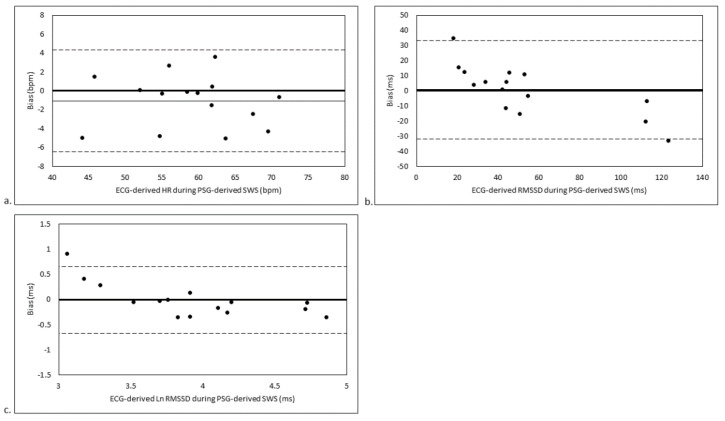
Agreement between (**a**) ECG-derived HR during PSG-derived SWS and WHOOP-derived HR during WHOOP-derived SWS, (**b**) ECG-derived RMSSD during PSG-derived SWS and WHOOP-derived RMSSD during WHOOP-derived SWS, and (**c**) ECG-derived Ln RMSSD during PSG-derived SWS and WHOOP-derived Ln RMSSD during WHOOP-derived SWS. Bias is calculated as WHOOP-derived variable during WHOOP-derived SWS minus ECG-derived variable during PSG-derived SWS. Thin continuous line represents mean bias. Dashed lines represent mean bias ± limits of agreement. bpm, beats per minute; ECG, electrocardiogram; HR, heart rate; Ln RMSSD, natural logarithm of the root mean square of successive BB interval differences; ms, milliseconds; PSG, polysomnography; RMSSD, root mean square of successive BB interval differences; SWS, slow-wave sleep.

For the analysis of agreement between ECG-derived HR/HRV during PSG-derived SWS and WHOOP-derived HR/HRV during WHOOP-derived SWS, there were trivial to small biases in HR (ES = 0.13 ± 0.17; 1.85 ± 2.51%; *p* = 0.15; Figure 2a) and HRV (ES = 0.03 ± 0.29; 20.08 ± 28.42%; *p* = 0.86 for RMSSD [Figure 2b] and ES = 0.21 ± 0.39; 3.74 ± 5.76%; *p* = 0.29 for Ln RMSSD [Figure 2c]). There were moderate to large LOAs for HR (9.71%; ES = 0.67), RMSSD (110.07%; ES = 1.12) and Ln RMSSD (22.31%; ES = 1.50).

**Figure 3:**
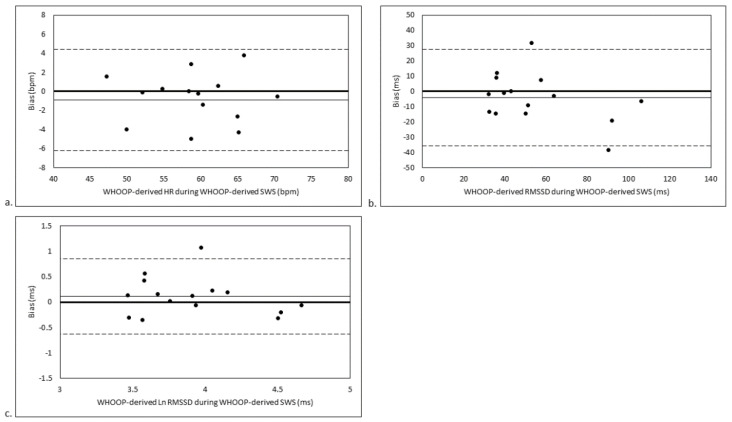
Agreement between (**a**) WHOOP-derived HR during PSG-derived SWS and WHOOP-derived HR during WHOOP-derived SWS, (**b**) WHOOP-derived RMSSD during PSG-derived SWS and WHOOP-derived RMSSD during WHOOP-derived SWS, and (**c**) WHOOP-derived Ln RMSSD during PSG-derived SWS and WHOOP-derived Ln RMSSD during WHOOP-derived SWS. Bias is calculated as WHOOP-derived variable during WHOOP-derived SWS minus WHOOP-derived variable during PSG-derived SWS. Thin continuous line represents mean bias. Dashed lines represent mean bias ± limits of agreement. bpm, beats per minute; HR, heart rate; Ln RMSSD, natural logarithm of the root mean square of successive BB interval differences; ms, milliseconds; PSG, polysomnography; RMSSD, root mean square of successive BB interval differences; SWS, slow-wave sleep.

For the analysis of agreement between WHOOP-derived HR/HRV during PSG-derived SWS and WHOOP-derived HR/HRV during WHOOP-derived SWS, there were trivial biases in HR (ES = 0.11 ± 0.17; 1.57 ± 2.45%; *p* = 0.21; Figure 3a) and HRV (ES = 0.16 ± 0.31; 5.75 ± 23.16%; *p* = 0.34 for RMSSD [Figure 3b] and ES = 0.01 ± 0.38; 0.68 ± 5.13%; *p* = 0.94 for Ln RMSSD [Figure 3c]). There were moderate to large LOAs for HR (9.48%; ES = 0.66), RMSSD (89.70%; ES = 1.21) and Ln RMSSD (19.85%; ES = 1.47).

**Figure 4:**
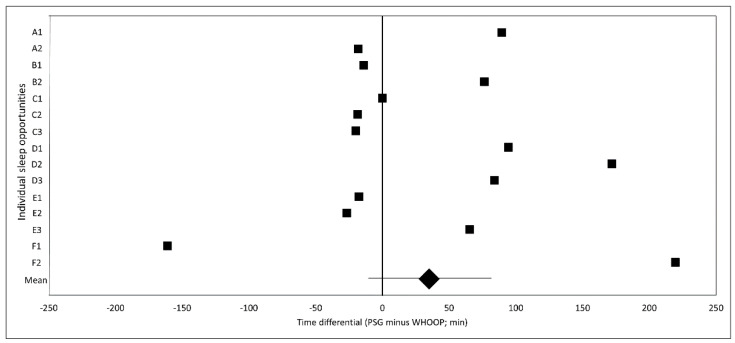
Time differential (min) for SWS periods derived by PSG and WHOOP. Data are presented as WHOOP time of day minus PSG time of day such that positive values indicate WHOOP-derived SWS periods occurred after PSG-derived SWS periods. Diamond marker represents mean bias ±95% confidence interval. Y-axis letters represent individual participants. Y-axis numerals represent individual participants’ sleep opportunities. min, minutes; PSG, polysomnography; SWS, slow-wave sleep.

Figure 4 depicts the time differential between WHOOP-derived SWS episodes and PSG-derived SWS episodes per sleep opportunity; there was a small bias (WHOOP minus PSG) of 35.1 ± 47.2 min (ES = 0.31 ± 0.42; *p* = 0.17) with large LOA of 182.7 min (ES = 1.62).

## 4. Discussion

This study evaluated agreement between PPG assessment of HR and HRV by a wearable biosensor (WHOOP 2.0) and gold-standard assessment via ECG. The primary findings were that of trivial bias (ES ≤ 0.03) and LOA (ES ≤ 0.10) for time-matched HR assessment, and trivial bias (ES ≤ 0.19) and small LOA (ES ≤ 0.59) for time-matched HRV assessment when either a Strong filter or WHOOP’s proprietary filter was applied to WHOOP-derived PP interval data and analysed as Ln RMSSD. SWS stage-matched WHOOP-derived HR and HRV demonstrated trivial bias (ES ≤ 0.11) and moderate to large LOA (ES = 0.66–1.47).

The present study identified that WHOOP-derived PP intervals need to be filtered prior to HRV calculation to facilitate optimal agreement with ECG-derived HRV. The methodological consideration of BB interval editing is not a novel concept and has been advocated in HRV analysis [2,5]. Indeed, Buchheit [5] demonstrated that a single erroneous BB interval over a five-minute recording substantially altered RMSSD calculation. Consequently, it is important to edit BB intervals prior to analysis to ensure a true reflection of ANS status. While visual inspection and manual editing of BB intervals is ideal, it is an unrealistic practice in the field where a multitude of files are recorded, and instantaneous feedback is required to guide athletic training. Accordingly, automatic BB interval editing within manufacture software is commonplace [5]. In the present study, bias and LOA was minimised, while ICC was maximised, as filter strength increased to a Strong level (and also with WHOOP’s proprietary filter). However, a Very Strong filter resulted in poorer agreement between WHOOP- and ECG-derived HRV, indicating this filter was too aggressive, and excessively altered the true BB interval patterning. Thus, the present study indicates that while erroneous WHOOP-derived PP intervals have little impact on HR calculation, these intervals have a small to moderate impact on RMSSD/Ln RMSSD calculation, as evidenced by the small biases (ES = 0.21–0.32) and small to moderate LOAs (ES = 0.56–0.78) in unfiltered RMSSD and Ln RMSSD across both SWS quantification methods, in comparison to the trivial biases (ES = 0.04–0.19) and small LOAs (ES = 0.33–0.59) in filtered values (i.e., Kubios’s Strong filter and WHOOP’s proprietary filter).

A further methodological consideration in HRV determination is the natural logarithm transformation of RMSSD (i.e., Ln RMSSD). Natural logarithm transformation reduces bias from non-uniformity of error [25], and has become standard practice for the longitudinal monitoring of training status via HRV [5]. In the present study, natural logarithm transformation of RMSSD resulted in small to moderate (ES = 0.56–1.19) reductions in percent bias, and large to very large (ES = 1.23–2.15) reductions in percent residuals in comparison to raw RMSSD.

While some statistically significant differences in bias were found between Kubios’s Strong filter and WHOOP’s proprietary filter, agreement statistics (i.e., bias and LOA) in WHOOP-derived HR and HRV may also be contextualised using the natural day-to-day variability in these variables. Some variation exists in the literature with regard to day-to-day variability in HR (10–11% coefficient of variation [5,26]) and Ln RMSSD (3–13% coefficient of variation [5,26,27,28,29,30,31,32]) which is likely dependent on timing of assessment (i.e., morning waking versus nocturnal) and posture (i.e., supine versus sitting versus standing). With specific regard to nocturnally collected Ln RMSSD, Costa et al. [32] demonstrated a coefficient of variation of 4–6%. Additionally, given that a smallest worthwhile change in HR/HRV has been proposed to be calculated as 0.5 multiplied by coefficient of variation [25], the smallest worthwhile change is 5–5.5% for HR and 1.5–6.5% for Ln RMSSD (and 2–3% for nocturnally derived Ln RMSSD). Consequently, since the bias (<0.5%) and LOA (1–1.5%) in WHOOP-derived HR were less than both the smallest worthwhile change and coefficient of variation in HR, it may be concluded that WHOOP’s proprietary filter provides suitable editing of PP intervals. However, since the bias (2–3.5%) and LOA (6–6.5%) in WHOOP-derived Ln RMSSD (when edited with WHOOP’s proprietary filter) approaches the upper limit for the smallest worthwhile change in Ln RMSSD (and exceeds both the coefficient of variation and smallest worthwhile change for nocturnally derived Ln RMSSD), WHOOP-derived measures of Ln RMSSD may need to be interpreted against their own level of bias precision (i.e., LOA of 6–6.5%).

The physiological determinants of the trivial biases with small LOAs for agreement between ECG- and PPG-derived Ln RMSSD demonstrated in the present study potentially lie within the pulse travel time. Specifically, the electrical activity of the heart is followed by spread of the pulsatile wave of blood to the periphery [12]. While this pulse travel time demonstrates BB fluctuations of only a few milliseconds [33,34,35], this does indicate that BB intervals derived from ECG and PPG will rarely be *exactly* the same, which intuitively indicates that variability in BB intervals (i.e., RMSSD/Ln RMSSD) will rarely be *exactly* the same also.

WHOOP quantify HR and HRV in the final SWS episode of each sleep opportunity, which is intuitive from a physiological perspective. SWS is thought to be important for physiological recovery from exercise [21], a hypothesis supported by the synchronisation between SWS periods and growth hormone release in humans (suggesting that sleep periods provide optimal anabolic conditions) and findings of SWS duration being proportional to preceding wakefulness [22]. Since HRV is a measure of ANS status [2], which is in turn considered a marker of physiological readiness to perform exercise [3], it is intuitive that HRV be assessed during SWS, since this may be used to quantify the magnitude of physiological recovery facilitated by SWS (although future research is required to confirm this). However, given that WHOOP has only moderate sensitivity in accurately identifying sleep stage [20], which is supported by the findings of the present study indicating a small time differential (ES = 0.31) in the determination of SWS periods by WHOOP in comparison to PSG (and a large [ES = 1.62] LOA about that time differential), the misrepresentation of true SWS periods by WHOOP may have an impact on WHOOP-derived HR and HRV since autonomic HR modulation is physiologically impacted upon by sleep stage [36,37,38,39]. Indeed, while the bias in stage-matched WHOOP-derived HR (ES = 0.11; 1.57%) and Ln RMSSD (ES = 0.01; 0.68%) during WHOOP-derived SWS episodes compared to PSG-derived SWS episodes was trivial, the LOA for WHOOP-derived HR (9.48%) exceeded the smallest worthwhile change (5–5.5%) and approached the coefficient of variation (10–11%) in this variable, while the LOA for WHOOP-derived Ln RMSSD (19.85%) exceeded both the smallest worthwhile change (1.5–6.5%) and coefficient of variation (3–13%) for this variable. Thus, while the exploratory analysis performed in the present study does not quantify the true day-to-day variability in WHOOP-derived HR and HRV, it does indicate that a misrepresentation of SWS periods impacts upon the precision of bias in WHOOP-derived HR and HRV, which may in turn impact upon the day-to-day variability in WHOOP-derived HR and HRV. Consequently, future research should evaluate the day-to-day variability in WHOOP-derived HR and HRV.

The agreement between PPG- and ECG-derived HR shown in this study (i.e., bias = 0.02–0.23 bpm [ES = 0.01–0.03]; LOA = 0.72–0.81 bpm; r = 1.00 across both SWS quantification methods) is similar to that previously shown in other wearable devices during quiet rest and sleep (bias = 0.09–1.00 bpm [ES = 0.01–0.09]; LOA = 2.67–5.29 bpm; r = 0.99) [8,10,11]. The agreement between wrist-based PPG- and ECG-derived HRV demonstrated in the present study (i.e., bias = 1.33–4.90 ms [ES = 0.04–0.14]; LOA = 11.26–15.96 ms; r = 0.98–0.99 for RMSSD) is within the range found using earlobe and fingertip PPG-derived HRV (bias = 0.00–2.71 ms [ES = 0.00–0.11]; LOA = 1.40–14.50 ms; r = 0.99–1.00) during quiet rest and sleep [7,12,13,14,15,16,17,18].

The present study used repeat measures in only a small number of unique individuals, which may be considered a limitation in certain scientific contexts. However, the authors propose this is not the case in the present study where simple statistical agreement is the focus. Specifically, to suitably “challenge” the WHOOP unit for accurately assessing PPG-derived HR and HRV, an appropriate range and variability in BB intervals is required. The current dataset provides HR in the range of 40–75 bpm and RMSSD in the range of 15–125 ms, and it is hypothesised that these ranges cover the typical range seen in both the general and athletic population.

While the WHOOP-derived HR and HRV variables assessed in the present study feed into WHOOP’s Recovery Score, validation of this Recovery Score itself was beyond the scope of this study, and thus should be validated in future research.

## 5. Conclusions

A wearable biosensor (WHOOP 2.0) demonstrated acceptable agreement in HR via PPG assessment compared with gold-standard assessment via ECG. Regarding HRV assessment however, bias and LOA in Ln RMSSD approached or exceeded the smallest worthwhile change/coefficient of variation for this variable, and thus should be interpreted against its own level of bias precision when suitably edited to remove erroneous PP intervals and analysed as Ln RMSSD. SWS stage-matched assessment of HR and HRV indicated that misrepresentation of SWS periods impacted upon the precision of bias in WHOOP-derived HR and HRV, which may in turn have an impact on the day-to-day variability in WHOOP-derived HR and HRV.

## Figures and Tables

**Table 1:** Analyses of agreement overview.

		HR/HRV Derived by…	
		WHOOP	ECG
SWS period derived by…	WHOOP	1,3,4	1
	PSG	2,4	2,3

HR, heart rate; HRV, heart rate variability; PSG, polysomnography; SWS, slow-wave sleep; 1, time-matched analysis of WHOOP-derived HR/HRV vs. ECG-derived HR/HRV during WHOOP-derived SWS; 2, time-matched analysis of WHOOP-derived HR/HRV vs. ECG-derived HR/HRV during PSG-derived SWS; 3, sleep stage-matched analysis of WHOOP-derived HR/HRV during WHOOP-derived SWS vs. ECG-derived HR/HRV during PSG-derived SWS; 4, sleep stage-matched analysis of WHOOP-derived HR/HRV during WHOOP-derived SWS vs. WHOOP-derived HR/HRV during PSG-derived SWS.

## Data Availability

The datasets generated from the current study are available from the corresponding author on reasonable request.

## Supplementary Materials

The following are available online at https://www.mdpi.com/1424-8220/21/10/3571/s1, Table S1. Agreement between ECG- and WHOOP-derived Heart Rate (bpm), Table S2. Agreement between ECG- and WHOOP-derived RMSSD (ms), Table S3. Agreement between ECG- and WHOOP-derived Ln RMSSD (ms).
