# Correction: Bellenger et al. Wrist-Based Photoplethysmography Assessment of Heart Rate and Heart Rate Variability: Validation of WHOOP. *Sensors* 2021, *21*, 3571

**DOI:** 10.3390/s22083090

**Published:** 2022-04-18

**Authors:** Clint R. Bellenger, Dean J. Miller, Shona L. Halson, Gregory D. Roach, Charli Sargent

**Affiliations:** 1 Alliance for Research in Exercise, Nutrition and Activity (ARENA), Allied Health and Human Performance, University of South Australia, Adelaide 5000, Australia; 2 South Australian Sports Institute, Adelaide 5000, Australia; 3 The Appleton Institute for Behavioural Science, Central Queensland University, Adelaide 5043, Australia; d.j.miller@cqu.edu.au (D.J.M.); greg.roach@cqu.edu.au (G.D.R.); charli.sargent@cqu.edu.au (C.S.); 4 School of Behavioural and Health Sciences, Australian Catholic University, Brisbane 4014, Australia; Shona.Halson@acu.edu.au

The authors wish to correct the following errors in the original paper [1].

## Author Name Correction

In the front matter, we added the abbreviation of middle name, instead of “Dean Miller”, it should be “Dean J. Miller”, instead of “Greg Roach”, it should be “Gregory D. Roach”.

## Affiliation Correction

In Affiliations 1 and 2, we updated the postcode, instead of “5001”, it should be “5000”. In Affiliation 3, we updated the postcode, instead of “5034”, it should be “5043”.

## Text Correction

- In the Abstract section on page 1, instead of “HR and HRV were assessed via WHOOP and ECG over 15 opportunities”, it should read “HR and HRV were assessed via WHOOP 2.0 and ECG over 15 opportunities during October–December 2018”.
- In the Introduction section on page 2, instead of “WHOOP is a commercially available unit that quantifies HR and HRV”, it should read “WHOOP 2.0 is a wearable biosensor that quantifies HR and HRV”.
- In the Experimental Overview sub-section of the Materials and Methods section on page 2, instead of “Data collection occurred concurrently with a larger pre-existing sleep study”, it should read “Data collection occurred in October–December 2018, concurrently with a larger pre-existing sleep study”.
- In the Experimental Overview sub-section of the Materials and Methods section on page 2, instead of “Participants wore a WHOOP unit”, it should read “Participants wore a WHOOP 2.0 unit”.
- In the Discussion section on page 8, instead of “This study evaluated agreement between PPG assessment of HR and HRV by a commercially available wrist-worn activity monitor (WHOOP) and gold-standard assessment via ECG”, it should read “This study evaluated agreement between PPG assessment of HR and HRV by a wearable biosensor (WHOOP 2.0) and gold-standard assessment via ECG”.
- In the Conclusions section on page 10, instead of “A wrist-worn, commercially available activity monitor (WHOOP) demonstrated acceptable agreement in HR via PPG assessment compared with gold-standard assessment via ECG”, it should read “A wearable biosensor (WHOOP 2.0) demonstrated acceptable agreement in HR via PPG assessment compared with gold-standard assessment via ECG”.

The authors apologize for any inconvenience caused and state that the scientific conclusions are unaffected. This correction was approved by the Academic Editor. The original publication has also been updated.
