# Supplementary material for: Wrist-Based Photoplethysmography Assessment of Heart Rate and Heart Rate Variability: Validation of WHOOP
Source: Sensors (Basel). 2021 May 20;21(10):3571. doi: 10.3390/s21103571 (PMC8160717; doi:10.3390/s21103571)
Supplement: Supplementary file 1 [file sensors-21-03571-s001.zip › sensors-1177213-supplementary/Supplementary Table 1 20210127.pdf]

Table 1. Agreement between ECG- and WHOOP-derived Heart Rate (bpm)

| Analysis | ECG | Agreement | Filter Strength | | | | | |
|---|---|---|---|---|---|---|---|---|
| | | | None | Low | Medium | Strong | Very Strong | WHOOP filter |
| Time matched; SWS detected by WHOOP | 58.06±4.16 | WHOOP | 58.04±4.14 | 58.03±4.14 | 58.04±4.13 | 58.03±4.13 | 58.00±4.15 | 57.82±4.12* |
| | | Bias | -0.02±0.18[w] | -0.02±0.18[w] | -0.02±0.19[w] | -0.02±0.19[w] | -0.05±0.19[w] | -0.23±0.21[n,l,m,s,vs] |
| | | Residual | 0.21±0.15[vs,w] | 0.22±0.14[w] | 0.22±0.14 | 0.22±0.15 | 0.24±0.14[n] | 0.27±0.15[n,l] |
| | | LOA | ±0.71 | ±0.71 | ±0.72 | ±0.72 | ±0.74 | ±0.81 |
| | | %Bias | -0.03±0.37[w] | -0.03±0.37[w] | -0.02±0.37[w] | -0.03±0.37[w] | -0.09±0.39[w] | -0.39±0.38[n,l,m,s,vs] |
| | | %Residual | 0.41±0.30[vs,w] | 0.42±0.30[w] | 0.43±0.30[w] | 0.42±0.30[w] | 0.47±0.30[n] | 0.52±0.30[n,l,m,s] |
| | | %LOA | ±1.43 | ±1.43 | ±1.44 | ±1.44 | ±1.52 | ±1.56 |
| | | ICC | 1.00 (0.99-1.00) | 1.00 (0.99-1.00) | 1.00 (0.99-1.00) | 1.00 (0.99-1.00) | 1.00 (0.99-1.00) | 1.00 (0.99-1.00) |
| Time matched; SWS detected by PSG | 58.90±3.98 | WHOOP | 58.93±4.00 | 58.93±4.00 | 58.93±4.00 | 58.93±3.99 | 58.88±4.02 | 58.74±4.03* |
| | | Bias | 0.03±0.12[w] | 0.03±0.12[w] | 0.03±0.12[w] | 0.03±0.12[w] | -0.03±0.14[w] | -0.16±0.13[n,l,m,s,vs] |
| | | Residual | 0.14±0.09 | 0.14±0.09 | 0.14±0.10 | 0.13±0.10 | 0.17±0.10 | 0.17±0.09 |
| | | LOA | ±0.46 | ±0.45 | ±0.48 | ±0.46 | ±0.53 | ±0.50 |
| | | %Bias | 0.04±0.22[w] | 0.04±0.22[w] | 0.04±0.23[w] | 0.05±0.22[w] | -0.06±0.25[w] | -0.29±0.24[n,l,m,s,vs] |
| | | %Residual | 0.25±0.17 | 0.25±0.17 | 0.25±0.19 | 0.23±0.18 | 0.32±0.19 | 0.33±0.16 |
| | | %LOA | ±0.84 | ±0.83 | ±0.89 | ±0.85 | ±0.97 | ±0.92 |
| | | ICC | 1.00 (0.99-1.00) | 1.00 (0.99-1.00) | 1.00 (0.99-1.00) | 1.00 (0.99-1.00) | 1.00 (0.99-1.00) | 1.00 (0.99-1.00) |

ECG, electrocardiogram; ICC, intraclass correlation coefficient; LOA, limits of agreement; PSG, polysomnography; SWS, slow wave sleep; *, significant difference between ECG- and WHOOP-derived HR ($P<0.05$); vs, significantly different from Very Strong filter ($P<0.05$); s, significantly different from Strong filter ($P<0.05$); m, significantly different from Medium filter ($P<0.05$); l, significantly different from Low filter ($P<0.05$); w, significantly different from WHOOP proprietary filter ($P<0.05$). Data are presented as mean ± 95% confidence interval. Bias is calculated as WHOOP value minus ECG value. %Bias is calculated as bias as a percentage of ECG value.
