# Supplementary material for: Wrist-Based Photoplethysmography Assessment of Heart Rate and Heart Rate Variability: Validation of WHOOP
Source: Sensors (Basel). 2021 May 20;21(10):3571. doi: 10.3390/s21103571 (PMC8160717; doi:10.3390/s21103571)
Supplement: Supplementary file 1 [file sensors-21-03571-s001.zip › sensors-1177213-supplementary/Supplementary Table 2 20210127.pdf]

Table 2. Agreement between ECG- and WHOOP-derived RMSSD (ms)

Analysis	ECG	Agreement	Filter Strength					
			None	Low	Medium	Strong	Very Strong	WHOOP filter
Time matched; SWS detected by WHOOP	51.27±12.16	WHOOP	56.09±12.22*	55.35±12.03*	54.3±11.65	53.02±11.14	46.92±7.84	54.47±12.00
		Bias	4.82±4.24^{vs}	4.07±3.65^{vs}	3.03±3.43^{vs}	1.75±3.53^{vs}	-4.35±5.81^{n,l,m,s,w}	3.20±3.57^{vs}
		Residual	5.07±3.30^{vs}	4.36±2.85^{vs}	4.21±2.63^{vs}	4.63±2.57^{vs}	9.87±2.66^{n,l,m,s,w}	4.33±2.75^{vs}
		LOA	±16.42	±14.14	±13.28	±13.68	±22.52	±13.81
		%Bias	11.78±7.81^{vs}	10.32±6.71^{vs}	8.54±6.64^{vs}	6.59±7.04^{vs}	-2.70±9.04^{n,l,m,s,w}	8.54±6.65^{vs}
		%Residual	10.97±5.28	9.41±4.55^{vs}	8.89±4.73^{vs}	10.45±4.42^{vs}	15.42±4.07^{l,m,s,w}	9.29±4.53^{vs}
		%LOA	±30.23	±25.98	±25.71	±27.26	±35.03	±25.75
		ICC	0.97 (0.91-0.99)	0.98 (0.93-0.99)	0.98 (0.94-0.99)	0.98 (0.93-0.99)	0.91 (0.74-0.97)	0.98 (0.93-0.99)
Time matched; SWS detected by PSG	53.69±17.37	WHOOP	61.73±16.56*	61.59±16.44*	58.59±16.62*	55.02±14.48	44.85±8.55	58.59±17.00*
		Bias	8.05±4.83^{s,vs}	7.91±4.87^{s,vs}	4.91±3.35^{s,vs}	1.33±4.12^{n,l,m,vs,w}	-8.83±10.63^{n,l,m,s,w}	4.90±2.91^{s,vs}
		Residual	6.70±3.32^{vs}	6.76±3.34^{vs}	4.26±2.50^{vs}	6.14±2.58^{vs}	15.77±6.70^{n,l,m,s,w}	3.85±2.09^{vs}
		LOA	±18.70	±18.85	±12.99	±15.96	±41.19	±11.26
		%Bias	23.37±17.93^{vs}	23.25±17.97^{vs}	13.25±7.45^{s,vs}	8.39±6.70^{m,vs,w}	-4.37±11.44^{n,l,m,s,w}	12.74±6.53^{s,vs}
		%Residual	21.17±14.09	21.22±14.12	10.41±5.08	11.25±3.18^{vs}	18.22±6.3^{s}	9.75±4.07
		%LOA	±69.43	±69.58	±28.87	±25.94	±44.29	±25.31
		ICC	0.98 (0.94-0.99)	0.98 (0.94-0.99)	0.99 (0.97-1.00)	0.98 (0.95-0.99)	0.82 (0.47-0.94)	0.99 (0.98-1.00)

ECG, electrocardiogram; ICC, intraclass correlation coefficient; LOA, limits of agreement; PSG, polysomnography; RMSSD, root mean square of successive beat to beat interval differences; SWS, slow wave sleep; *, significant difference between ECG- and WHOOP-derived RMSSD ($P<0.05$); vs, significantly different from Very Strong filter ($P<0.05$); s, significantly different from Strong filter ($P<0.05$); m, significantly different from Medium filter ($P<0.05$); l, significantly different from Low filter ($P<0.05$); w, significantly different from WHOOP proprietary filter ($P<0.05$). Data are presented as mean ± 95% confidence interval. Bias is calculated as WHOOP value minus ECG value. %Bias is calculated as bias as a percentage of ECG value.
