# Supplementary material for: Wrist-Based Photoplethysmography Assessment of Heart Rate and Heart Rate Variability: Validation of WHOOP
Source: Sensors (Basel). 2021 May 20;21(10):3571. doi: 10.3390/s21103571 (PMC8160717; doi:10.3390/s21103571)
Supplement: Supplementary file 1 [file sensors-21-03571-s001.zip › sensors-1177213-supplementary/Supplementary Table 3 20210127.pdf]

Table 3. Agreement between ECG- and WHOOP-derived Ln RMSSD (ms)

| Sleep stage | ECG | Agreement | Filter Strength | | | | | |
|---|---|---|---|---|---|---|---|---|
| | | | None | Low | Medium | Strong | Very Strong | WHOOP filter |
| Time matched; SWS detected by WHOOP | 3.85±0.22 | WHOOP | 3.95±0.20* | 3.94±0.20* | 3.92±0.20* | 3.90±0.19 | 3.80±0.16 | 3.92±0.20* |
| | | Bias | 0.10±0.07[vs] | 0.09±0.07[vs] | 0.07±0.07[vs] | 0.06±0.07[vs] | -0.04±0.10[n,l,m,s,w] | 0.07±0.07[vs] |
| | | Residual | 0.10±0.05[vs] | 0.09±0.05[vs] | 0.09±0.05[vs] | 0.10±0.04[vs] | 0.17±0.04[n,l,m,s,w] | 0.09±0.05[vs] |
| | | LOA | ±0.29 | ±0.26 | ±0.25 | ±0.27 | ±0.37 | ±0.25 |
| | | %Bias | 2.81±1.89[vs] | 2.53±1.71[vs] | 2.12±1.71[vs] | 1.66±1.80[vs] | -0.77±2.40[n,l,m,s,w] | 2.11±1.70[vs] |
| | | %Residual | 2.66±1.27[vs] | 2.35±1.18[vs] | 2.28±1.22[vs] | 2.69±1.12[vs] | 4.10±1.07[n,l,m,s,w] | 2.34±1.18[vs] |
| | | %LOA | ±7.30 | ±6.62 | ±6.61 | ±6.98 | ±9.30 | ±6.59 |
| | | ICC | 0.97 (0.91-0.99) | 0.97 (0.92-0.99) | 0.97 (0.92-0.99) | 0.97 (0.91-0.99) | 0.93 (0.79-0.98) | 0.98 (0.93-0.99) |
| Time matched; SWS detected by PSG | 3.81±0.30 | WHOOP | 4.00±0.27* | 3.99±0.27* | 3.93±0.28* | 3.89±0.26* | 3.74±0.19 | 3.93±0.28* |
| | | Bias | 0.18±0.11[s,vs] | 0.18±0.11[s,vs] | 0.12±0.06[s,vs] | 0.07±0.06[n,l,m,vs,w] | -0.08±0.14[n,l,m,s,w] | 0.11±0.06[s,vs] |
| | | Residual | 0.14±0.09 | 0.14±0.09 | 0.09±0.04[vs] | 0.11±0.03[vs] | 0.21±0.08[m,s,w] | 0.09±0.03[vs] |
| | | LOA | ±0.44 | ±0.44 | ±0.24 | ±0.25 | ±0.54 | ±0.21 |
| | | %Bias | 5.29±3.53[vs] | 5.27±3.54[vs] | 3.35±1.67[s,vs] | 2.30±1.69[m,vs,w] | -1.18±3.23[n,l,m,s,w] | 3.25±1.53[s,vs] |
| | | %Residual | 4.15±2.78 | 4.17±2.79 | 2.64±0.93[vs] | 2.88±0.76[vs] | 5.11±1.82[m,s,w] | 2.54±0.76[vs] |
| | | %LOA | ±13.68 | ±13.72 | ±6.45 | ±6.54 | ±12.53 | ±5.93 |
| | | ICC | 0.96 (0.88-0.99) | 0.96 (0.88-0.99) | 0.99 (0.97-1.00) | 0.99 (0.97-1.00) | 0.92 (0.76-0.97) | 0.99 (0.97-1.00) |

ECG, electrocardiogram; ICC, intraclass correlation coefficient; Ln RMSSD, natural logarithm of the root mean square of successive beat to beat interval differences; LOA, limits of agreement; PSG, polysomnography; SWS, slow wave sleep; *, significant difference between ECG- and WHOOP-derived Ln RMSSD (P<0.05); vs, significantly different from Very Strong filter (P<0.05); s, significantly different from Strong filter (P<0.05); m, significantly different from Medium filter (P<0.05); l, significantly different from Low filter (P<0.05); w, significantly different from WHOOP proprietary filter (P<0.05). Data are presented as mean ± 95% confidence interval. Bias is calculated as WHOOP value minus ECG value. %Bias is calculated as bias as a percentage of ECG value.
